# Two Dimensional Array of Piezoresistive Nanomechanical Membrane-Type Surface Stress Sensor(MSS) with Improved Sensitivity

**DOI:** 10.3390/s121115873

**Published:** 2012-11-16

**Authors:** Genki Yoshikawa, Terunobu Akiyama, Frederic Loizeau, Kota Shiba, Sebastian Gautsch, Tomonobu Nakayama, Peter Vettiger, Nico F. de Rooij, Masakazu Aono

**Affiliations:** 1 International Center for Materials Nanoarchitectonics (MANA), National Institute for Materials Science (NIMS), Tsukuba 305-0044, Japan; E-Mails: SHIBA.Kota@nims.go.jp (K.S.); NAKAYAMA.Tomonobu@nims.go.jp (T.N.); AONO.Masakazu@nims.go.jp (M.A.); 2 Institute of Microengineering (IMT), Ecole Polytechnique Fédérale de Lausanne (EPFL), Neuchâtel CH-2002, Switzerland; E-Mails: terunobu.akiyama@epfl.ch (T.A.); frederic.loizeau@epfl.ch (F.L.); sebastian.gautsch@epfl.ch (S.G.); pvettiger@bluewin.ch (P.V.); nico.derooij@epfl.ch (N.F.R.)

**Keywords:** Membrane-type Surface stress Sensor(MSS), nanomechanical sensors, piezoresistive, MEMS/NEMS

## Abstract

We present a new generation of piezoresistive nanomechanical Membrane-type Surface stress Sensor(MSS) chips, which consist of a two dimensional array of MSS on a single chip. The implementation of several optimization techniques in the design and microfabrication improved the piezoresistive sensitivity by 3∼4 times compared to the first generation MSS chip, resulting in a sensitivity about ∼100 times better than a standard cantilever-type sensor and a few times better than optical read-out methods in terms of experimental signal-to-noise ratio. Since the integrated piezoresistive read-out of the MSS can meet practical requirements, such as compactness and not requiring bulky and expensive peripheral devices, the MSS is a promising transducer for nanomechanical sensing in the rapidly growing application fields in medicine, biology, security, and the environment. Specifically, its system compactness due to the integrated piezoresistive sensing makes the MSS concept attractive for the instruments used in mobile applications. In addition, the MSS can operate in opaque liquids, such as blood, where optical read-out techniques cannot be applied.

## 1. Introduction

Since all molecules have “volume” and “mass”, transduction of such fundamental parameters into detectable and processable signals can realize label-free and real-time measurements of virtually any kind of target specimen. Nanomechanical sensors have been emerging as a key device employing this strategy with their multiple operation modes: static and dynamic modes which basically measure volume or mass via deflection or resonance frequency shift, respectively [1,2]. In this study, we focus on the static mode, as it does not suffer from damping effects because the gradual bending motion caused by the analyte-induced surface stress is usually slow enough not to be affected by damping. In addition, the scope of the surface stress-based sensing can cover various phenomena because surface stress emerges not only with the adsorption of molecules but also with the nanomechanical structural changes of substances on the surface [3,4].

Cantilevers are well known as a representative example among various geometries of nanomechanical sensors [4–11]. Most studies employ optical (laser) measurement of cantilever deflection [1,2,4–11], but this approach has several practical problems. One of the most promising solutions to these problems is employing lever-integrated piezoresistive sensing [12–18] as it does not require bulky and complex peripheral devices related with an optical read-out and can also be used for measurements in opaque liquids, such as blood. In spite of these inherent advantages, piezoresistive cantilevers have not been widely used for sensing applications because of their low sensitivity compared to optical read-out cantilevers.

**Figure 1.**
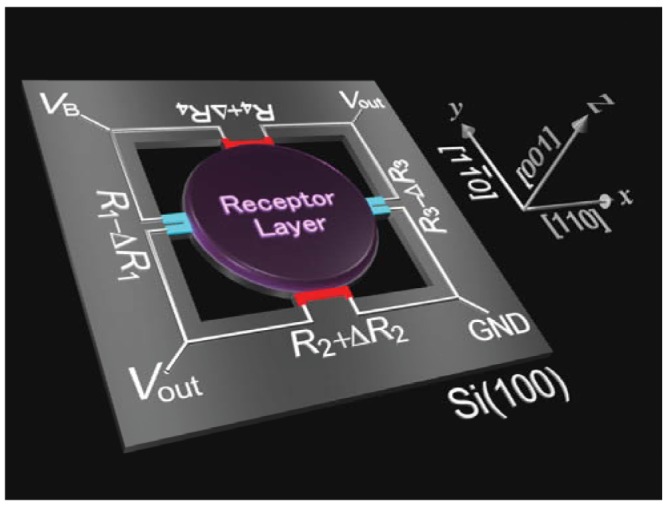
Schematic illustration of the MSS. The whole surface stress induced on the round center membrane is efficiently detected by piezoresistors (red and blue colored parts) embedded in the constricted beams. Note that the new MSS chip fabricated in the present study has a different arrangement of piezoresistors on the sensing beams, which is discussed in Section 2.4.

To improve the sensitivity for integrated piezoresistive sensors, we recently made a comprehensive structural optimization of a piezoresistive cantilever. Breaking the bounds of common practice of a “cantilever”, we developed a membrane-type surface stress sensor (MSS) which consists of an “adsorbate membrane” supported with four constrictions called “sensing beams”, on which piezoresistors are integrated (Figure 1) [19]. With this configuration, the analyte-induced isotropic surface stress on the receptor-coated membrane is efficiently transduced onto the sensing beams as a uniaxial stress amplified by the constriction and, thus, piezoresistors embedded at these sensing beams can efficiently detect the whole surface stress applied on the adsorbate membrane. The piezoresistors of the four sensing beams are used to form a full Wheatstone bridge configuration in which all four resistors generate pairs of resistance changes in opposite signs. This configuration improves the sensitivity further by a factor of ∼4 and it enhances the thermal stability by self-compensation. With the first prototype MSS chip, we experimentally demonstrated a higher sensitivity which was a factor of 20 more than that of a standard piezoresistive cantilever and thus was comparable to that of optical read-out cantilevers [19].

While the first generation prototype MSS (1G-MSS) chips with one dimensional (1D) MSS arrays were fabricated as a proof-of-concept of MSS and succeeded in demonstrating its significantly high sensitivity, further improvement in sensitivity is important for providing further opportunities for MSS to be utilized in various applications. In addition, multi-dimensional arrays are also required to arrange a large number of sensing elements on a single chip for the simultaneous detection of a wide variety of analytes.

**Figure 2.**
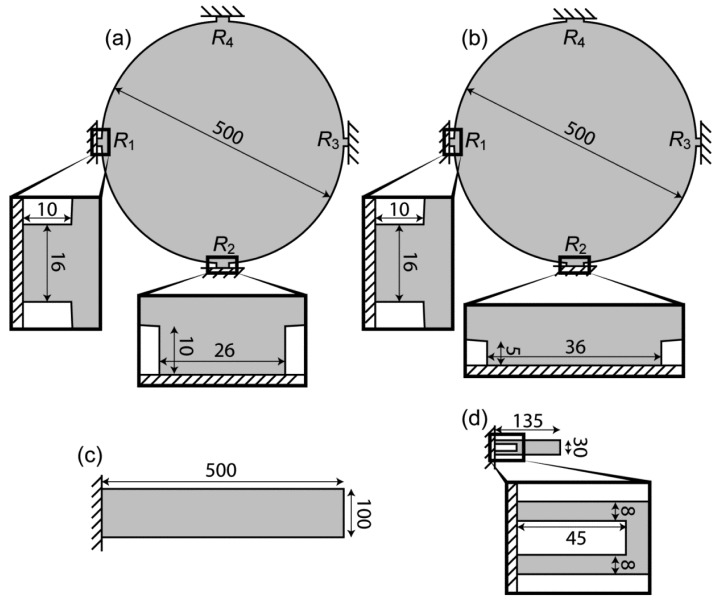
Dimensions of the sensors which are compared in the present study. All figures are illustrated in top-view. Numbers indicate the dimensions in μm. (**a**) 2G-MSS (thickness 2.5 μm); (**b**) 1G-MSS (thickness 3.2 μm); (**c**) optically read-out cantilever (thickness 1 μm); (**d**) piezoresistive cantilever (thickness 1 μm) [14]. The piezoresistors-integrated parts are magnified in the insets. Note that *R*_1_, *R*_3_ and *R*_2_, *R*_4_ in the 2G-MSS and 1G-MSS have the same dimensions, respectively.

In the present study, we fabricated the second generation MSS (2G-MSS) chip, which has a two dimensional (2D) array of MSS on a single chip, consisting of nine channels (3 × 3). We have also modified several components of MSS to further improve the sensitivity. The sensors which we compare in the present study are illustrated in Figure 2 with their dimensions. The technical improvements in design and microfabrication significantly improved the sensitivity of 2G-MSS (Figure 2(a)), achieving 3∼4 times higher sensitivity than the 1G-MSS (Figure 2(b)) or an optical read-out cantilever (Figure 2(c)) and exceeding ∼100 times the sensitivity of a standard piezoresistive cantilever-type sensor (Figure 2(d)) [14]. Within the system, the MSS chip can be inserted into a standard 0.5 mm pitch connector; this eliminates a wire-bonding procedure, offering easy chip exchange, and provides all required electrical input/output connections. The MSS can detect almost any kind of molecule under various conditions, such as vacuum, gas, and liquid; including opaque liquids like blood. In addition, it can be miniaturized, integrated, and mass-produced owing to its CMOS compatibility, which fulfill most requirements for realizing a portable sensing device. Given the various conveniences and advantages of the integrated piezoresistive read-out with the significantly improved sensitivity, MSS is expected to find practical applications in various fields.

## 2. Improvement in Sensitivity

According to the analytical considerations on various geometries of nanomechanical sensors [20] and the know-how on microfabrication developed so far [13–15,17,21,22], we made several modifications on each part of the MSS design as follows:

1. Narrower sensing beams with thinner geometry
2. Thinner passivation layers on piezoresistors
3. Shallower piezoresistors with lower doping level
4. Elimination of negative regions in piezoresistors

We will discuss each modification in detail in the following sections. To confirm each contribution quantitatively, finite element analysis (FEA) was performed using COMSOL Multiphysics 3.5a with the Structural Mechanics module. Each structure was meshed with 10,000∼20,000 elements, which give sufficient resolution for the present simulations. In all cases, the surface stress was set at −3.0 N/m, which was simulated by applying initial stress (−3.0 × 10^8^ Pa) on a thin film (10 nm) covered on the top surface of the membrane based on the analytically verified assumption that the surface stress (*s*) can be described using the thickness of a thin enough film on the surface (*t_surf_*) and the three-dimensional “bulk” stress (*σ_bulk_*) applied in the thin film [19,23]: *s* = *σ_bulk_* · *t_surf_*. The Young's moduli and Poisson's ratios of each material were set as follows: Si[170 GPa, 0.28], SiO_2_[70 GPa, 0.17], and Si_3_N_4_[250 GPa, 0.23]. The averaged values of relative resistance change (Δ*R*/*R*|_ave_) on the plane with the area of *A*_R_ at the middle position of piezoresistors (see Section 2.3 for details) in the piezoresistive sensing beams are used to estimate the performance:

(1) $${\frac{\Delta R}{R}|}_{\text{ave}}=\frac{1}{A_{\text{R}}}\int_{0}^{A_{\text{R}}}\frac{\Delta R}{R}\text{dA}$$

Note that, in the following sections, the distribution of Δ*R*/*R* is simulated assuming that the current flows in the [110] direction. These relative resistance changes are valid only for the piezoresistors (*i.e.*, boron-doped p-type region), while non-doped regions do not have such resistance changes. See also Section 2.4 for details.

### 2.1. Narrower Sensing Beams with Thinner Geometry

**Figure 3.**
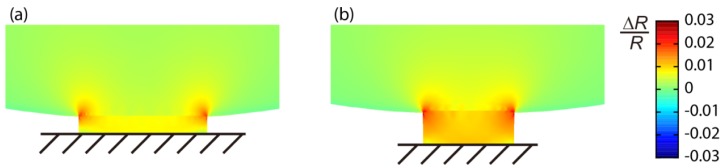
FEA of the distribution of Δ*R*/*R* on the *R*_2_ beams of (**a**) 1G-MSS and (**b**) 2G-MSS. Beam sizes: (length × width × thickness): 1G-MSS chip 5 × 36 × 3.2 μm^3^; 2G-MSS chip 10 × 26 × 2.5 μm^3^. In this simulation, only the structural components, e.g., the membrane and beams, are included. Note that the legend is valid for both (a) and (b) and is also the case for Figures 4 and 5.

The important difference between the MSS and a standard piezoresistive cantilever is the size dependence on a signal. In the MSS geometry, the stresses on the piezoresistors are basically determined by the aspect ratio between a membrane and beams, whereas there is almost no dependence in the case of standard cantilever geometry [19]. Thus, to achieve a larger signal, we should make either the adsorbate membrane larger or the sensing beams smaller. Since the former strategy will increase the size of the chip as well as the absolute amount of analytes required to gain the same coverage on the surface, we made the smaller sensing beams; more specifically, narrower sensing beams. We also found with FEA that the stress applied on the sensing beams increases almost linearly by decreasing the thickness of the whole geometry including the membrane and beams. Figure 3 shows the results of FEA on the distribution of Δ*R*/*R* induced on the sensing beams of two different sizes of the *R*_2_ beam for both 1G- and 2G-MSS chips. The sizes for each beam are as follows (length × width × thickness): 1G-MSS chip 5 × 36 × 3.2 μm^3^; 2G-MSS chip 10 × 26 × 2.5 μm^3^. According to the FEA, the thinner geometry with smaller beams in the 2G-MSS chip enhances the signal by about 40%.

### 2.2. Thinner Passivation Layers on Piezoresistors

**Figure 4.**
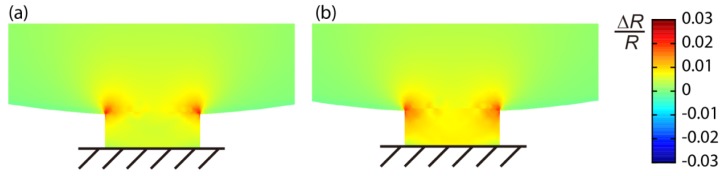
FEA of the distribution of Δ*R*/*R* on the *R*_2_ beams of the 2G-MSS chip passivated by (**a**) 650 nm of SiO_2_ and 100 nm of Si_3_N_4_ and (**b**) 80 nm of SiO_2_ and 80 nm of Si_3_N_4_.

Passivation is one of the most important techniques for piezoresistor-based devices to prevent electric leakage in various environments, especially in liquid media. However, there is a trade-off between the quality of protection and sensitivity; a thicker passivation layer provides better quality of protection, while a thinner layer increases flexibility, leading to higher sensitivity of the piezoresistor. To achieve both the protection quality and sensitivity, we deposited a thin silicon nitride (Si_3_N_4_) film by low pressure chemical vapor deposition (LPCVD), as we demonstrated its high protection quality even at rather small thicknesses [14]. While the 1G-MSS chip was passivated by CVD SiO_2_ (650 nm) and LPCVD Si_3_N_4_ (100 nm), thermal oxide SiO_2_ (80 nm) and LPCVD Si_3_N_4_ (80 nm) were deposited on the piezoresistors of 2G-MSS chips. Figure 4 shows the FEA of 2G-MSS chips coated by the thick (SiO_2_ 650 nm + Si_3_N_4_ 100 nm) and thin (SiO_2_ 80 nm + Si_3_N_4_ 80 nm) passivation layers. It is found that the thinner passivation layer of Si_3_N_4_ improves the sensitivity by about 40%.

We also investigated the stability of the thin Si_3_N_4_ passivation layer in liquid environments. The 2G-MSS chip was confirmed to have no electric leakage for at least a week even in a conductive solution like phosphate buffered saline (PBS), indicating its long-term stability, which is essential for various applications.

### 2.3. Shallower Piezoresistors with Lower Doping Level

**Figure 5.**
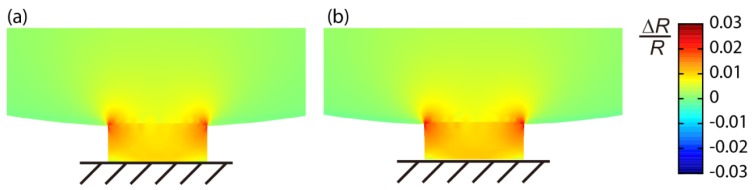
FEA of the distribution of Δ*R*/*R* on the *R*_2_ beams of the 2G-MSS chip at (**a**) 250 nm and (**b**) 150 nm below the surface, respectively.

According to basic mechanics [24], the stress (*σ*) induced in a bending beam is given as follows:

(2) $$\sigma =\frac{E}{R}z$$

where *E* is the Young's modulus of the beam, *R* is the radius of bending, and *z* is the distance from the neutral axis, along which there is no compression or dilation. In the case of constant bending, the stress is proportional to the distance *z* from the neutral axis and reaches maximum at the surface of the beam. Thus, piezoresistors closer to the surface yield larger stress, resulting in higher sensitivity. This simple mechanics can be also confirmed by FEA as shown in Figure 5. In the present study, piezoresistors were made by boron implantation, forming ∼300 nm of doped region from the surface of each beam in the chip. On the other hand, the piezoresistors in the 1G-MSS chip had ∼500 nm of doped region made by boron diffusion. Assuming that the current flows along the middle region of each piezoresistor, the shallower doping in the 2G-MSS chip improves the sensitivity by about ∼10%. It should be noted that the implantation results in lower doping level than the diffusion method. Since the piezoresistance factor increases as the impurity concentration decreases [25], the piezoresistors made by implantation in 2G-MSS should gain additional amplification in sensitivity.

### 2.4. Elimination of Negative Regions in Piezoresistors

In the design of a piezoresistive transducer, the direction of current flow is of critical importance. To simplify the discussion, we focus on Si(100), which we used for fabricating the MSS chips. The piezoresistors made in the present study are boron-doped *p*-type regions, while the other regions are *n*-type. The relation between the relative resistance change (Δ*R*/*R*) and stress (*σ*) can be described as follows [26]:

(3) $$\frac{\Delta R}{R}=\sum \pi_{\text{cs}}\sigma_{s}$$

where *π_cs_* denotes a piezoresistance coefficient with the current flow and stress in the directions indicated by the subscriptions “*c*” and “*s*”, respectively, e.g., “*π_xy_*” corresponds to the piezoresistance coefficient with the current flowing along *x* direction and stress induced in *y* direction. *σ_s_* is the stress induced in the “*s*” direction. Here we define *x* and *y* directions as [11̄0] and [10] for *p*-type Si(100) as illustrated in Figure 1. In this case, the relevant piezoresistance coefficients are calculated as follows [26]:

(4) $$\pi_{\text{xx}}=\pi_{\text{yy}}=\frac{1}{2}\left(\pi_{11}+\pi_{12}+\pi_{44}\right)\sim \frac{1}{2}\pi_{44}$$

(5) $$\pi_{\text{xy}}=\pi_{\text{yx}}=\frac{1}{2}\left(\pi_{11}+\pi_{12}-\pi_{44}\right)\sim -\frac{1}{2}\pi_{44}$$

where *π*_11_, *π*_12_, and *π*_44_ are the fundamental piezoresistance coefficients of the silicon crystal; for *p*-type Si(100), their values are estimated as +6.6, −1.1, and +138.1 in units of 10^−11^ Pa^−1^, respectively [25]. Thus, in the cases of current flowing in *x* or *y* direction, the relative resistance change in each case is given by the following Equations (6) or (7):

(6) $$\frac{\Delta R_{x}}{R_{x}}=\pi_{\text{xx}}\sigma_{x}+\pi_{\text{xy}}\sigma_{y}\sim \frac{1}{2}\pi_{44}\sigma_{x}-\frac{1}{2}\pi_{44}\sigma_{y}=\frac{1}{2}\pi_{44}(\sigma_{x}-\sigma_{y})$$

(7) $$\frac{\Delta R_{y}}{R_{y}}=\pi_{\text{yx}}\sigma_{x}+\pi_{\text{yy}}\sigma_{y}\sim -\frac{1}{2}\pi_{44}\sigma_{x}+\frac{1}{2}\pi_{44}\sigma_{y}=-\frac{1}{2}\pi_{44}(\sigma_{x}-\sigma_{y})$$

**Figure 6.**
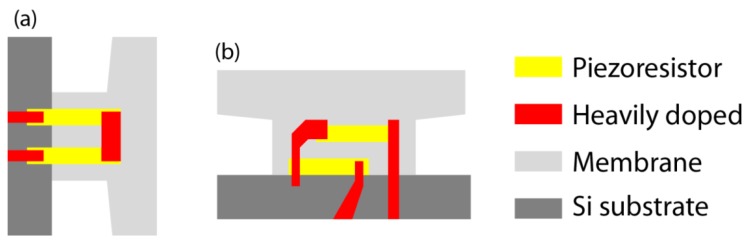
The piezoresistors on (**a**) *R*_1_ and (**b**) *R*_2_ in the 2G-MSS chip.

These equations indicate that the coexistence of mutually perpendicular current flows in the domain with a stress induced in the same direction gives rise to a negative contribution to the gross relative resistance change of the domain. Since the dominant stresses in each constricted beam in the MSS are *σ_x_* (≫*σ_y_*) for *R*_1_ and *R*_3_ and *σ_y_* (≫*σ_x_*) for *R_2_* and *R*_4_, the current flow should be fixed in *x* direction for all sensing beams to gain the maximum output of the full Wheatstone bridge which is approximately given by:

(8) $$V_{\text{out}}=\frac{V_{\text{B}}}{4}\left(\frac{\Delta R_{1}}{R_{1}}-\frac{\Delta R_{2}}{R_{2}}+\frac{\Delta R_{3}}{R_{3}}-\frac{\Delta R_{4}}{R_{4}}\right)$$

where *V*_B_ is bias voltage applied to the bridge. To avoid the negatively contributing region, we designed the piezoresistors as shown in Figure 6. The perpendicular current paths are replaced by the heavily doped regions which simply make the electrical connection of the piezoresistors. Based on the simple calculation, the elimination of the negative contribution region improves the sensitivity by about ∼30%.

### 2.5. Overall Improvement

The improvements in each component are summarized in Table 1. Accounting for all contributions, the sensitivity of the new MSS chip is improved by a factor of ∼3 compared to the 1G-MSS chip. Since the FEA of the 1G-MSS chip indicates ∼43 times higher sensitivity compared to the standard piezoresistive cantilever [19], the 2G-MSS has been found to have ∼130 times higher sensitivity than the standard piezoresistive cantilever. It should be taken into account that the modifications implemented in the 2G-MSS chip mutually affect each other and the effects which are not included in the present FEA can have some influence on the overall sensitivity. Note that the sensitivity of the MSS structure can be tuned by changing the dimensions of each component, e.g., the larger aspect ratio between the membrane and beams leads to a larger enhancement of sensitivity, whereas there is almost no dependence for the standard cantilever architecture. Because of this property, one can design one's own MSS depending on their required parameters for sensitivity, size, robustness, and the amount of analyte available.

### 2.6. Signal-to-Noise Ratio (S/N_exp_)

The modifications of piezoresistors lead to the changes in the intrinsic noises; Johnson (thermal) noise (*V_J_*) and Hooge (1/*f*) noise (*V_H_*)). These noises can be estimated by the following equations [19,27–29]:

(9) $$\bar{{V_{J}}^{2}}=4k_{B}T\frac{l_{p}}{w_{p}t_{p}\mu qp}(f_{\max}-f_{\min})$$

(10) $$\bar{{V_{H}}^{2}}=\frac{\alpha {V_{B}}^{2}}{l_{p}w_{p}t_{p}p}\ln\left(\frac{f_{\max}}{f_{\min}}\right)$$

Thus, the total noise in the piezoresistors (*V_Total_*) can be calculated as follows:

(11) $$\begin{array}{cc} V_{\text{Total}} & =\sqrt{\bar{{V_{J}}^{2}}+\bar{{V_{H}}^{2}}}\\ & =\sqrt{4k_{B}T\frac{l_{p}}{w_{p}t_{p}\mu qp}(f_{\max}-f_{\min})+\frac{\alpha {V_{B}}^{2}}{l_{p}w_{p}t_{p}p}\ln\left(\frac{f_{\max}}{f_{\min}}\right)} \end{array}$$

**Figure 7.**
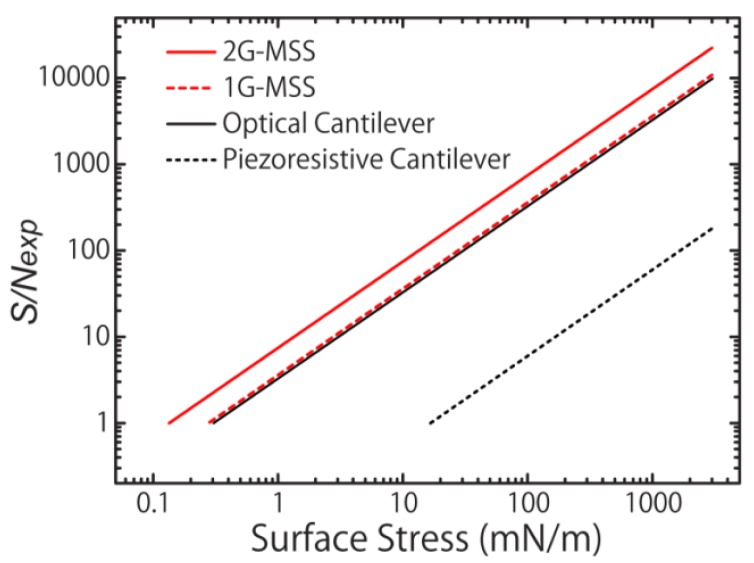
Experimental signal-to-noise ratio (*S/N_exp_*) of 2G-MSS (red solid line), 1G-MSS (red dashed line), the optical cantilever (black solid line), and the piezoresistive cantilever (black dashed line) as a function of the induced surface stress. The experimental noises (*N_exp_*); 1.0 μV and 1 nm for piezoresistive and optical read-outs, respectively.

For a full Wheatstone bridge, this total noise value is doubled [30]. The descriptions and values of the constants are summarized in Table 2. According to these estimations, it is found that the intrinsic noises are still lower than the experimental noises (*N_exp_*; 1.0∼1.5 μV), which were recorded in the present measurements (see details in Section 4) and were mainly caused by the electrical circuit. On the other hand, the experimental noises (*N_exp_*) for the optically read-out cantilevers have been reported as 0.5∼3 nm in the previous studies [5,7–11,31]. Based on these values, the experimental signal-to-noise levels (*S/N_exp_*) are plotted in Figure 7 as a function of the induced surface stress. For a simple comparison, the experimental noise levels for piezoresistive and optical read-outs are set at 1.0 μV and 1 nm, respectively. The minimum detectable surface stress for each sensor corresponds to the points around *S*/*N_exp_*∼1. Note that this figure is based on the assumption that the signals of all sensors increase linearly as the induced surface stress increases. The factors that are not included in the FEA will affect the values, especially in the cases of extreme conditions; for example, a very large surface stress will result in the deviation from Equation (8). In such a case, one would also take account of the fracture stress of silicon.

## 3. Fabrication of MSS in 2D Array

The 2G-MSS chip with MSS arranged in 2D array was fabricated by the following procedure: 4-inch silicon-on-insulator (SOI) wafers with a *n*-type device layer (∼4 μm-thick, ∼10 Ω·cm) were prepared. After cleaning the wafers, 700 nm of oxide was formed by thermal oxidation. The highly doped diffusion area was patterned into the oxide by photolithography and etching in buffered HF (BHF). A double layer (650 nm-thick) of boron-doped and non-doped silicon oxide was deposited by chemical vapor deposition (CVD). The diffusion of boron was carried out at 950 °C for 30 min in nitrogen. Oxide on the membrane area was removed and a new thermal oxide was grown. Using a patterned photo resist layer as a mask, boron implantation was performed. After a rapid thermal annealing (RTA), a silicon nitride layer was deposited by LPCVD on the entire wafer surface. Following a photolithography, contact holes were opened by plasma and BHF etching. An aluminum layer of 900 nm was evaporated on the device side. It was patterned in a chemical etchant with delineated photoresist mask. To isolate the electrodes from analytes in device operation, plasma enhanced CVD was performed to form a 700 nm-thick oxide. Pad openings were made by photolithography and chemical etching. Etching windows on the back side of the wafer for KOH etching were formed. The wafer was then mounted in a mechanical check and immersed in KOH at 40 weight % at 60°C to form large openings from the back side. The etching automatically stopped at the buried oxide (BOX) layer of SOI. Finally, the BOX was etched in BHF and the membrane was released.

**Figure 8.**
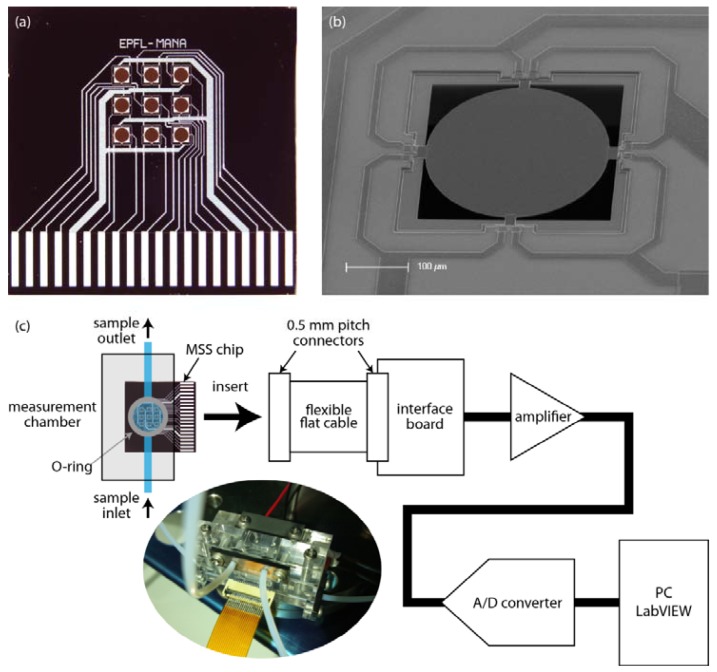
(**a**) Photograph of the fabricated 2G-MSS chip with a 2D array. This chip can be ready for use just by inserting the chip into a standard 0.5 mm pitch connector for an electrical connection without any additional procedure. (**b**) Enlarged SEM image of one of the MSS elements in an array of the 2G-MSS chip. (**c**) An example of MSS measurement system diagram. (Inset) Photograph of a 2G-MSS chip connected with a standard 0.5 mm pitch connector and mounted in a measurement chamber with O-rings for sealing.

The fabricated 2G-MSS chip is presented in Figure 8(a). There are nine channels (3 × 3) in the 2D array. All nine of the channels as well as electrodes for bridge bias voltage and ground are connected to the 0.5 mm-pitch pads at the bottom for the electrical connection to an external read-out device (e.g., amplifier and analog-digital converter) without any addition procedure, such as wire-bonding. The scanning electron microscope (SEM) image shows an enlarged image of the 2G-MSS (Figure 8(b)).

## 4. Experimental Verification

**Figure 9.**
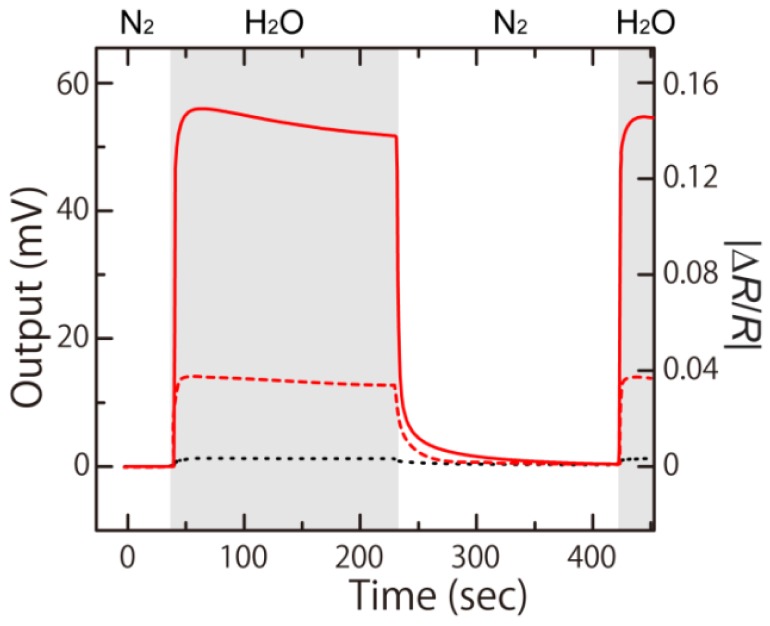
Obtained output signals (*V*_out_) from the 2G-MSS chip (red solid line), 1G-MSS chip (red dashed line), and standard piezoresistive cantilever (black dotted line).

To verify the technological improvements implemented in the 2G-MSS, we experimentally assessed the performance of the newly fabricated chip shown in Figure 8. The circular membrane was coated with poly(sodium 4-styrenesulfonate) (PSS) layers by a customized ink-jet spotting system (Microjet Co. Ltd., Model ‘LaboJet-500SP’). PSS solution with a concentration of 5 g/L was deposited to form a 1 μm thick PSS layer on the surface. The PSS-coated MSS chip was mounted in a closed chamber and was exposed to 20% water vapor in pure nitrogen carrier gas with a flow rate of 100 mL/min for 3 min followed by nitrogen purging for 3 min. Figure 9 shows the obtained output signal (*V*_out_) of the 2G-MSS chip compared to a 1G-MSS chip and a standard piezoresistive cantilever. The signals were measured with the same electrical setup with a bias voltage of −1.5 V in all cases. The 2G-MSS chip exhibited ∼4 and ∼100 times larger signals than those of the 1G-MSS chip and the standard piezoresistive cantilever, respectively. These experimental results confirm a significantly enhanced sensitivity of the 2G-MSS chip. The discrepancy from the FEA is presumably due to some effects which are not included in the present FEA; e.g., mutual effects of each modification, distribution of Δ*R*/*R* and the position of piezoresistors on the sensing beams, piezoresistance factor which is determined by the doping level [25], actual current paths in the piezoresistors, and the properties of the coating layers [32].

## 5. Conclusions/Outlook

We have fabricated a new MSS chip with a 2D array exhibiting improved sensitivity. The implementation of various modifications in design and microfabrication led to further sensitivity enhancements, reaching more than ∼100 times higher sensitivity compared to that of the standard piezoresistive cantilever. Based on the fact that the 1G-MSS chip was already demonstrated to have comparable sensitivity to the optical read-out standard cantilevers [19], the 2G-MSS has exceeded the optically read-out cantilevers in terms of experimental signal-to-noise ratio (*S*/*N_exp_*). It means that the MSS platform can cover most of applications so far demonstrated by the optically read-out cantilever sensors for surface stress-based sensing (*i.e.*, static mode). Owing to the size dependence in an MSS structure, one can modify the geometrical parameters to obtain even higher sensitivity. Since the MSS measures surface stress affected by the volume-based steric repulsion of adsorbed analyte molecules, it can detect almost any kind of molecule under various conditions including liquid, gas, and vacuum environments. In addition, it can be miniaturized with simple architecture at low cost owing to its CMOS compatibility to realize portable sensor devices, and also can be used in opaque liquids, such as blood. Given such conveniences and advantages of the integrated piezoresistive read-out, the MSS is one of the most promising transducers for nanomechanical sensors, offering various possibilities of practical applications in medicine, security, and environment fields.

## Figures and Tables

**Table 1.** Summary of the modifications made in the present study and the improvement in sensitivity compared to the 1G-MSS.

**Modifications**	**Improvement in Sensitivity**
(1) Narrower sensing beams with thinner geometry	∼40%
(2) Thinner passivation layers on piezoresistors	∼40%
(3) Shallower piezoresistors with lower doping level	∼10%
(4) Elimination of negative regions in piezoresistors	∼30%
Total	∼280%

**Table 2.** Summary of the descriptions and values of the constants related to the intrinsic noises. The calculated total noises (*V_Total_*) for 2G-MSS, 1G-MSS, and the piezoresistive cantilever are given at the bottom row.

**Description**	**Symbol**	**Unit**	**Value**		

			**2G-MSS**	**1G-MSS**	**Piezoresistive cantilever**
Length of piezoresistor	*l_p_*	cm	20 × 10^−4^	20 × 10^−4^	90 × 10^−4^
Width of piezoresistor	*w_p_*	cm	3.8 × 10^−4^	3.8 × 10^−4^	8 × 10^−4^
Thickness of piezoresistor	*t_p_*	cm	0.3 × 10^−4^	0.5 × 10^−4^	0.1 × 10^−4^
Carrier density	*p*	cm^−3^	4 × 10^18^	8 × 10^19^	8 × 10^19^
Carrier mobility	*μ*	cm^2^/Vs	100	50	50
Hooge constant	*α*	-		10^6^	
Maximum frequency	*f*_max_	Hz		3	
Minimum frequency	*f*_min_	Hz		0.1	
Bridge bias voltage	*V_B_*	V		1.5	
Temperature	*T*	K		293	
Boltzmann constant	*k_B_*	J/K		1.38 × 10^−23^	
Electron charge	*q*	C		1.60 × 10^−19^	
Total noise	*V_Total_*	V	6.37 × 10^−7^	1.01 × 10^−7^	3.76 × 10^−8^

## References

[1] Gimzewski J.K., Gerber C., Meyer E., Schlittler R.R. (1994). Observation of a chemical-reaction using a micromechanical sensor. Chem. Phys. Lett..

[2] Thundat T., Warmack R.J., Chen G.Y., Allison D.P. (1994). Thermal and ambient-induced deflections of scanning force microscope cantilevers. Appl. Phys. Lett..

[3] Mukhopadhyay R., Sumbayev V.V., Lorentzen M., Kjems J., Andreasen P.A., Besenbacher F. (2005). Cantilever sensor for nanomechanical detection of specific protein conformations. Nano Lett..

[4] Ghatkesar M.K., Lang H.P., Gerber C., Hegner M., Braun T. (2008). Comprehensive characterization of molecular interactions based on nanomechanics. PLoS One.

[5] Backmann N., Zahnd C., Huber F., Bietsch A., Pluckthun A., Lang H.P., Güntherodt H.J., Hegner M., Gerber C. (2005). A label-free immunosensor array using single-chain antibody fragments. Proc. Natl. Acad. Sci. USA.

[6] Berger R., Delamarche E., Lang H.P., Gerber C., Gimzewski J.K., Meyer E., Guntherodt H.J. (1997). Surface stress in the self-assembly of alkanethiols on gold. Science.

[7] Fritz J., Baller M.K., Lang H.P., Rothuizen H., Vettiger P., Meyer E., Guntherodt H.J., Gerber C., Gimzewski J.K. (2000). Translating biomolecular recognition into nanomechanics. Science.

[8] Lang H.P., Baller M.K., Berger R., Gerber C., Gimzewski J.K., Battiston F.M., Fornaro P., Ramseyer J.P., Meyer E., Güntherodt H.J. (1999). An artificial nose based on a micromechanical cantilever array. Anal. Chim. Acta.

[9] McKendry R., Zhang J.Y., Arntz Y., Strunz T., Hegner M., Lang H.P., Baller M.K., Certa U., Meyer E., Güntherodt H.J., Gerber C. (2002). Multiple label-free biodetection and quantitative DNA-binding assays on a nanomechanical cantilever array. Proc. Natl. Acad. Sci. USA.

[10] Watari M., Galbraith J., Lang H.P., Sousa M., Hegner M., Gerber C., Horton M.A., McKendry R.A. (2007). Investigating the molecular mechanisms of in-plane mechanochemistry on cantilever arrays. J. Am. Chem. Soc..

[11] Zhang J., Lang H.P., Huber F., Bietsch A., Grange W., Certa U., McKendry R., Güntherodt H.-J., Hegner M., Gerber C. (2006). Rapid and label-free nanomechanical detection of biomarker transcripts in human RNA. Nat. Nanotechnol..

[12] Tortonese M., Barrett R.C., Quate C.F. (1993). Atomic resolution with an atomic force microscope using piezoresistive detection. Appl. Phys. Lett..

[13] Akiyama T., Gautsch S., de Rooij N.F., Staufer U., Niedermann P., Howald L., Muller D., Tonin A., Hidber H.R., Pike W.T. (2001). Atomic force microscope for planetary applications. Sens. Actuators A.

[14] Aeschimann L., Meister A., Akiyama T., Chui B.W., Niedermann P., Heinzelmann H., De Rooij N.F., Staufer U., Vettiger P. (2006). Scanning probe arrays for life sciences and nanobiology applications. Microelectron. Eng..

[15] Yoshikawa G., Lang H.P., Akiyama T., Aeschimann L., Staufer U., Vettiger P., Aono M., Sakurai T., Gerber C. (2009). Sub-ppm detection of vapors using piezoresistive microcantilever array sensors. Nanotechnology.

[16] Boisen A., Thundat T. (2009). Design & fabrication of cantilever array biosensors. Mater. Today.

[17] Gautsch S., Akiyama T., Imer R., de Rooij N.F., Staufer U., Niedermann P., Howald L., Brandlin D., Tonin A., Hidber H.R., Pike W.T. (2002). Measurement of quartz particles by means of an atomic force microscope for planetary exploration. Surf. Interface Anal..

[18] Hierlemann A., Lange D., Hagleitner C., Kerness N., Koll A., Brand O., Baltes H. (2000). Application-specific sensor systems based on CMOS chemical microsensors. Sens. Actuators B.

[19] Yoshikawa G., Akiyama T., Gautsch S., Vettiger P., Rohrer H. (2011). Nanomechanical membrane-type surface stress sensor. Nano Lett..

[20] Yoshikawa G., Rohrer H. Strain Amplification Schemes for Piezoresistive Cantilevers.

[21] Lutwyche M., Andreoli C., Binnig G., Brugger J., Drechsler U., Haberle W., Rohrer H., Rothuizen H., Vettiger P., Yaralioglu G. (1999). 5X5 2D AFM cantilever arrays a first step towards a Terabit storage device. Sens. Actuators A.

[22] Hecht M.H., Marshall J., Pike W.T., Staufer U., Blaney D., Braendlin D., Gautsch S., Goetz W., Hidber H.R., Keller H.U. (2008). Microscopy capabilities of the microscopy, electrochemistry, and conductivity analyzer. J. Geophys. Res-Planet..

[23] Ricci A., Giuri E., Ricciardi C. Simulation of Surface Stress Effect on Mechanical Behavior of Silicon Microcantilevers.

[24] Sarid D. (1994). Scanning Force Microscopy.

[25] Kanda Y. (1982). A graphical representation of the piezoresistance coefficients in silicon. IEEE Trans. Electron. Dev..

[26] Pfann W.G., Thurston R.N. (1961). Semiconducting stress transducers utilizing transverse and shear piezoresistance effects. J. Appl. Phys..

[27] Harley J.A., Kenny T.W. (2000). 1/F noise considerations for the design and process optimization of piezoresistive cantilevers. J. Microelectromech. Syst..

[28] Yu X.M., Thaysen J., Hansen O., Boisen A. (2002). Optimization of sensitivity and noise in piezoresistive cantilevers. J. Appl. Phys..

[29] Hooge F.N. (1969). 1/F Noise is no surface effect. Phys. Lett. A.

[30] Mallon J.R., Rastegar A.J., Barlian A.A., Meyer M.T., Fung T.H., Pruitt B.L. (2008). Low 1/f noise, full bridge, microcantilever with longitudinal and transverse piezoresistors. Appl. Phys. Lett..

[31] Ndieyira J.W., Watari M., Barrera A.D., Zhou D., Vogtli M., Batchelor M., Cooper M.A., Strunz T., Horton M.A., Abell C. (2008). Nanomechanical detection of antibiotic mucopeptide binding in a model for superbug drug resistance. Nat. Nanotechnol..

[32] Yoshikawa G. (2011). Mechanical analysis and optimization of a microcantilever sensor coated with a solid receptor film. Appl. Phys. Lett..

